# Serum miR-125b is a non-invasive predictive biomarker of the pre-operative chemoradiotherapy responsiveness in patients with rectal adenocarcinoma

**DOI:** 10.18632/oncotarget.8725

**Published:** 2016-04-13

**Authors:** Edoardo D'Angelo, Matteo Fassan, Isacco Maretto, Salvatore Pucciarelli, Carlo Zanon, Maura Digito, Massimo Rugge, Donato Nitti, Marco Agostini

**Affiliations:** ^1^ Department of Surgical, Oncological and Gastroenterological Sciences, University of Padua, Padua, Italy; ^2^ Nanoinspired Biomedicine Laboratory, Institute of Pediatric Research, Fondazione Città della Speranza, Padua, Italy; ^3^ Department of Medicine (DIMED), Surgical Pathology & Cytopathology Unit, University of Padua, Padua, Italy; ^4^ Neuroblastoma Laboratory, Pediatric Research Institute, Città della Speranza, Padua, Italy; ^5^ Department of Nanomedicine, The Methodist Hospital Research Institute, Houston, Texas, USA

**Keywords:** rectal cancer, microRNA, miR-125b, circulating, pre-operative chemoradiotherapy

## Abstract

**Background:**

Therapeutic management of Locally Advanced Rectal Cancer (LARC) involves pre-operative chemoradiotherapy (pCRT) followed by surgery. However, after pCRT the complete pathological response is approximately 20%, whereas in 20 to 40% of patients the response is poor or absent.

**Methods:**

Cancer biopsy specimens (n= 38) and serum samples (n= 34) obtained before pCRT from 38 LARC patients were included in the study. Patients were classified in responders (R, tumor regression grade [TRG] 1-2; n= 16) and non-responders (NR, TRG 3-5; n= 22) according to the pathological response observed upon surgery. We performed miRNA microarrays analysis on biopsy specimens, and validated the selected candidates both by qRT-PCR (tissue and serum) and by *in situ* hybridization (tissue, miR-125b) analyses.

**Results:**

Eleven miRNAs were significantly different between R and NR (miR-154, miR-409-3p, miR-127-3p, miR-214*, miR-299-5p and miR-125b overexpressed in NR; miR-33a, miR-30e, miR-338-3p, miR-200a and miR-378 decreased). In particular, miR-125b resulted to be the best candidate to discriminate the two groups (AUC of 0.9026; 95% CI, 0.7618-1.043). Additionally, miR-125b serum levels were significantly overexpressed in NR patients compared to R (p-value=0.0087), with an excellent discriminating power (AUC of 0.782; 95% CI, 0.6123-0.9518).

**Conclusions:**

The obtained results further support the clinical impact of miRNA analysis. High miR-125b expression in tissue and serum were associated with a poor treatment response in LARC patients, therefore miR-125b could be considered as a possible novel non-invasive biomarker of response in LARC treatment.

## INTRODUCTION

Colorectal cancer (CRC) is the third leading cause of cancer death, both in men and women in the United States [1]. According to the American Cancer Society epidemiological data, 132, 700 new CRC cases have been diagnosed in 2015 [1]. Among CRCs, rectal cancer (RC) represents the 28% of the cases, and approximately 40% of RC are locally advanced at diagnosis (Locally Advanced Rectal Cancer [LARC]; T3, T4 or N+) [2].

The therapeutic management of LARC has deeply changed in the last forty years, and in contrast to non-rectal CRC, typically involves pre-operative chemoradiotherapy (pCRT) followed by surgery [3]. Several retrospective analyses suggest that higher tumor stages after neoadjuvant treatment have significant prognostic impact on both disease-free and overall survival [4–6]. In spite of this relevant clinical implication, only 20% of the cases present complete pathological response after pCRT, whereas in 20 to 40% of the patients, the response is poor or absent [7]. Thus, the identification of reliable and non-invasive predictive markers of cancer response to pCRT have a crucial importance.

MicroRNAs (miRNAs) are a class of small, non-coding RNAs raging in size from 19 to 25 nucleotides and act as post-transcriptional regulators of genes expression, by silencing their 3′-UTR-mRNA targets [8, 9]. Several studies demonstrated that this class of small molecules plays an important role in a variety of biologic processes including proliferation, apoptosis and cell differentiation, that are known to be deregulated in human malignancies [10, 11]. In particular, aberrant miRNA expression has been consistently reported in a vast number of tumor histotypes, including RC [12–22] and some miRNAs profiles have been associated to pCRT tumor response [23–28]. However, the current studies in the field are still unclear and controversial.

In a significant amount of mono-institutional series of LARC patients, we tested the prognostic impact of miRNAs expression in subjects resulted responders and non-responders to pCRT. In this study, we combined two specific miRNAs key features: *i)* the growing proofs of their involvement in determining anticancer-drug treatment response [27, 29–33]; *ii)* their high resistance to degradation and, as consequence, their easy detection in the body fluids [34–36].

## RESULTS

### Clinical-pathological characteristics of the considered LARC series

In order to identify a specific molecular signature of the clinical response to pCRT, we analyzed the miRNAs expression profile of 38 patients affected by rectal adenocarcinoma. The clinical data of the 38 patients are reported in Table 1. There was a prevalence of males (25 males *vs* 13 females) and the median age was 64 years. The median tumor distance from the anal verge was 7.5 cm (range 2-11 cm). The majority of the patients were clinically defined at the cTNM III stage (n=35), whereas the remaining three patients at the cTNM II stage. The baseline CEA level was ≥ 5 ng/ml in only 2 out of 38 patients.

**Table 1:** Clinical-pathological characteristics of 38 LARC patients

Patient	R/ NR	Gender	Age	AC	CEA	cTNM	Type of Surgery	Chemotherapy	Administration	RT Dose	ypT	ypN	ypM	ypTNM	TRG	OS	DFS
**E149**	R	W	43	8	1.2	3	LAR	CAP	OS	5500	0	0	0	0	1	6	6
**E82**	R	M	48	8	0.8	3	LAR	5-FU + OXA	IC	5040	0	0	0	0	1	58	58
**E109**	R	M	59	7	0.5	3	LAR	5-FU	IC	5040	0	0	0	0	1	30	30
**E110**	R	W	72	8	0.7	3	EL	CAP	OS	5500	0	X	X	X	1	28	28
**E133**	R	W	78	9	3	3	LAR	CAP	OS	5500	0	0	0	0	1	18	18
**E138**	R	M	72	10	0.5	3	EL	5-FU + OXA	OS	5040	0	X	X	X	1	17	17
**E114**	R	M	65	8	4.1	3	LAR	5-FU + CAP	OS	5500	0	0	0	0	1	23	23
**E124**	R	W	60	4	1.5	3	LAR	CAP + OXA	OS	5040	1	0	0	1	2	38	38
**E135**	R	M	54	8	0.9	3	LAR	CAP + OXA	OS	5040	1	0	0	1	2	19	19
**E139**	R	W	66	8	0.5	3	LAR	CAP	OS	5500	1	0	0	1	2	17	17
**E101**	R	W	73	10	0.6	2	LAR	CAP + OXA	OS	5040	Tis	0	0	0	1	38	38
**E113**	R	M	63	11	2.4	3	LAR	CAP	OS	4500	2	0	0	1	2	24	24
**E137**	R	W	56	8	0.5	3	LAR	CAP	OS	5500	2	0	0	1	2	18	9
**E141**	R	M	77	6	2.6	3	LAR	CAP + OXA	OS	5040	2	0	0	1	2	13	13
**E130**	R	M	68	4	0.8	2	LAR	CAP	OS	5500	2	0	0	1	2	26	26
**E100**	R	W	73	3	5.2	3	APR	5-FU + OXA	IC	5040	4	0	0	2	2	39	39
**E142**	NR	M	69	10	2.3	3	EL	CAP	OS	5500	2	X	0	X	3	21	21
**E143**	NR	M	64	8	1.7	3	LAR	CAP	OS	5500	3	0	0	2	3	22	22
**E146**	NR	M	64	6	0.9	3	LAR	CAP + OXA	OS	5040	3	0	0	2	4	7	7
**E151**	NR	W	68	11	1.2	3	APR	CAP + OXA	OS	5040	4	1	0	3	3	15	15
**E102**	NR	M	61	4	1.3	3	LAR	5-FU	IC	5040	3	0	0	2	3	42	42
**E126**	NR	W	61	5	0.8	3	LAR	CAP + OXA	OS	3060	1	0	0	1	4	51	51
**E98**	NR	M	59	10	0.5	3	LAR	CAP + OXA	OS	5040	2	0	0	1	3	42	42
**E125**	NR	M	58	4	0.1	3	CAA	CAP	OS	5500	2	0	1	4	5	41	41
**E123**	NR	M	54	5	1.7	3	CAA	CAP	OS	5500	2	0	0	1	3	27	7
**E91**	NR	M	69	8	1.1	3	LAR	CAP	OS	5500	3	0	0	2	4	24	24
**E94**	NR	M	62	6	10	3	LAR	CAP + OXA	OS	4500	3	0	0	2	3	50	5
**E99**	NR	W	62	2	1.2	3	APR	5-FU	IC	5040	3	0	0	2	3	43	43
**E105**	NR	M	68	5	1.8	3	LAR	5-FU + OXA	IC	5040	3	0	0	2	3	41	41
**E103**	NR	M	72	10	1.4	3	LAR	CAP + OXA	OS	5040	3	0	0	2	3	41	41
**E108**	NR	M	61	5	1.1	3	LAR	CAP	OS	5500	3	1	0	3	4	37	37
**E107**	NR	M	63	3	2.4	3	CAA	5-FU + OXA	IC	5040	3	0	0	2	3	22	12
**E111**	NR	M	66	5	0.9	3	LAR	CAP + OXA	OS	5040	3	1	0	3	3	25	25
**E132**	NR	M	79	6	1.2	3	LAR	5-FU	OS	5040	3	2	0	3	3	22	22
**E134**	NR	M	57	9	1.4	3	LAR	5-FU + OXA	OS	5040	3	0	0	2	4	20	20
**E129**	NR	M	50	11	1.3	3	LAR	5-FU + OXA	OS	5040	3	1	0	3	3	21	21
**E131**	NR	W	74	6	1.2	2	LAR	CAP	OS	5500	3	0	0	2	3	17	7
**E140**	NR	W	73	4	1.3	3	LAR	CAP	OS	5500	3	1	0	3	3	15	15

Abbreviations: AC: Centimeters from anal verge; CEA: carcinoembryonic antigen; cTNM: clinical TNM stage; LAR: low anterior resection; APR: abdominoperineal resection; CAA: Ultralow anterior resection with colon-anal anastomosis; EL: Local Excision; 5-FU: 5-Fluorouracil; ypT: pathological T stage after neoadjuvant treatments; Tis: Carcinoma *in situ*; TRG: tumor regression grade; X: undetermined value; DFS: Disease Free Survival; OS: Overall Survival.

The detailed characteristics of the treatment are listed in Table 1. A radical tumor resection was performed in all patients, except for three of them that had local excision. For these three patients, although surgical radical margins were achieved, the pathological lymph node status remained undefined. The surgical procedure specifically applied in this study was the Lower Anterior Resection (LAR) in combination with Total Mesorectal Excision (TME) (76.3%).

Patients were classified as responders (R) and non-responders (NR) according to Mandard TRG classification [37]: 16 (42%) were responders (TRG 1-2) and 22 (58%) non-responders (TRG 3-4-5). Eight patients (21%) showed a pathological complete response (ypT0 or TRG 1).

### A specific microRNAs signature discriminate pCRT responders from non-responders patients

**Figure 1:**
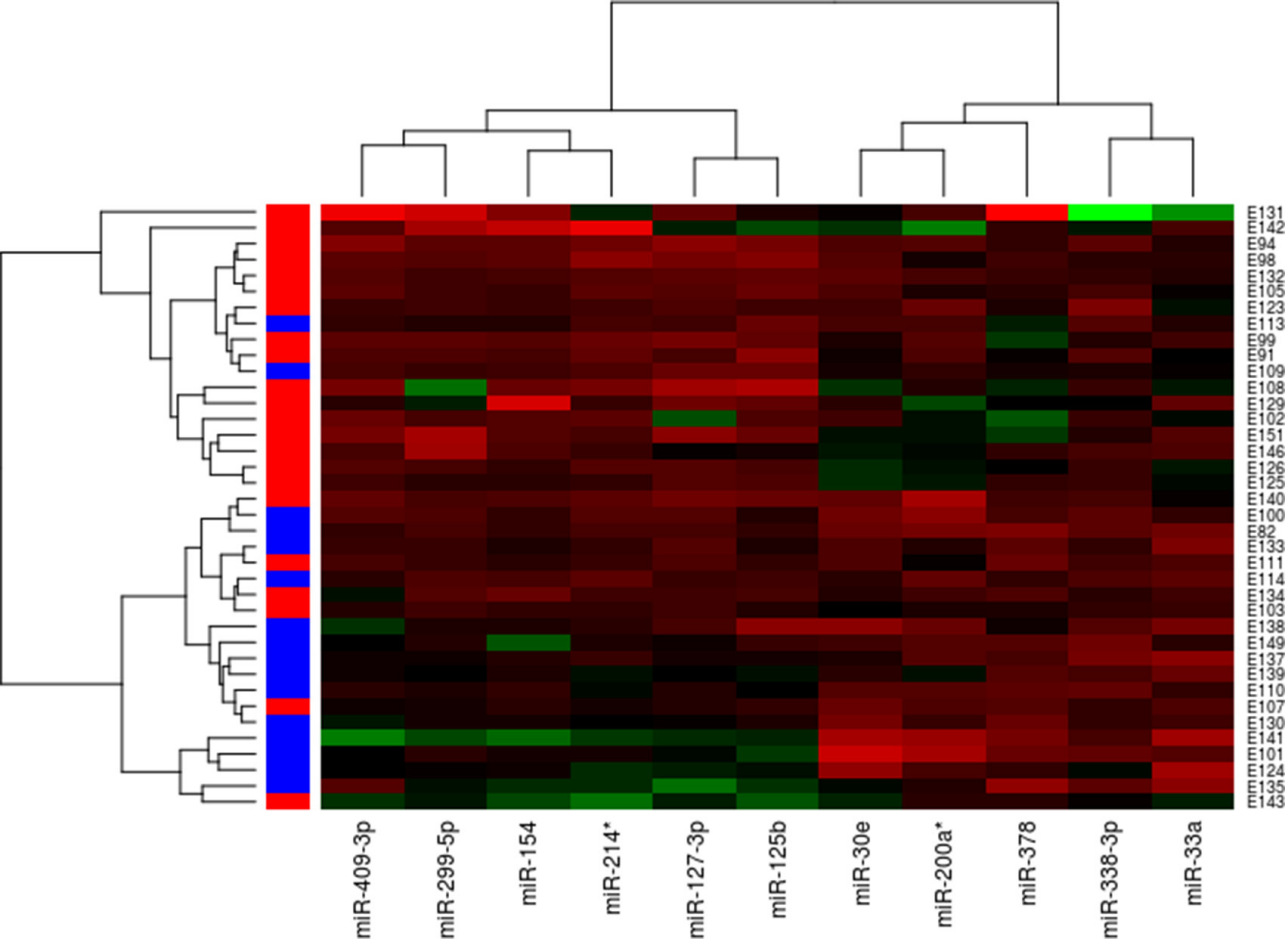
Hierarchical clustering of 38 LARC patients based on 11 differentially expressed miRNAs between responders and non-responders to pCRT Responders are in blue, Non-responders are in red. Red indicate an increased gene expression; green indicates a decreased expression.

Large-scale miRNA expression analysis was performed on 38 tissue samples of pretreatment pre-operative LARC biopsy samples. The ratio between tumor sample expression and the mean of normal samples expression was measured. Unsupervised hierarchical clustering analysis using the Ward clustering method and the Euclidean distance with a 5% FDR were performed. The preliminary analysis identified 11 miRNAs having a specific different expression pattern in NR and R patients. Among these, miR-154, miR-409-3p, miR-127-3p, miR-214*, miR-299-5p and miR-125b were overexpressed in NR patients, whereas miR-33a, miR-30e, miR-338-3p, miR-200a and miR-378, showed a lower expression levels. The results are summarized in the hierarchical clustering map in Figure 1. Single miRNAs normalized ratio expression level and p-value are reported in the Supplementary Figure S1.

**Figure 2:**
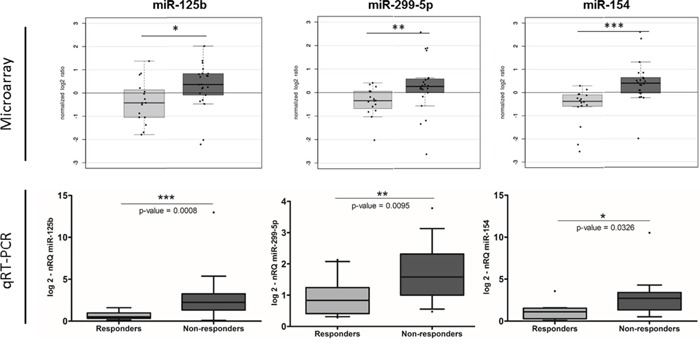
Tissue miR-125b, miR-299-5p and miR-154 expression levels were evaluated firstly by microarray and then confirmed by qRT-PCR (nRQ, normalized Relative Quantity– Responders, light grey; Non-responders, dark grey)

To confirm the predictive microRNA signature obtained from the microarray analysis, using the same RNA samples, we performed qRT-PCR for three selected miRNAs using the same RNA extracted from pCRT biopsies used in microarray analysis. Two miRNAs candidate (miR-299-5p and miR-154) were among the most significantly altered in microarray analysis; the other one, miR-125b, it has been previously associated to pCRT tumor response in other cancer settings [38] [39] [40]. qRT-PCR results sustained what observed in the microarray analysis. Indeed, the expression level of the all three selected miRNAs was highly different between the two groups and the one of miR-125b was the most significant (miR-125b, p-value=0.0008; miR-299-5p, p-value=0.0095; miR-154, p-value=0.0326) (Figure 2).

### Tissue miR-125b expression level distinguish between responders and non-responders patients with LARC

**Figure 3:**
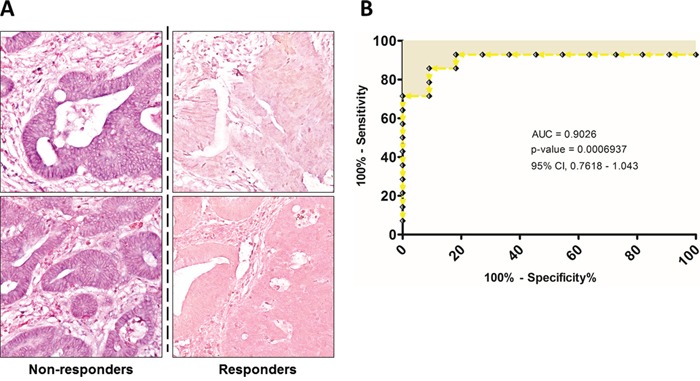
**A.** Representative ISH evaluation of miR-125b in responders and non-responders rectal cancers. The presence of miR-125b is shown by a grainy blue cytoplasmic stain; the slides are counterstained in fast red. (Original magnifications 20x). **B.** ROC curve analysis were performed based on tissue miR-125b expression in order to evaluate its efficiency on correctly classifying patients according to treatment response.

To further support the miR-125b expression results described above, we decided to perform tissue *in situ* hybridization (ISH) analysis. Among all the 38 biopsies, only 8 samples (4 responders and 4 non-responders patients) were suitable for ISH studies. As highlighted in Figure 3A, a consistent significant overexpression of miR-125b was observed in NR patients compared to R. The miR-125b was highly expressed in the cytoplasm of cancerous epithelial cells and characterized by a granular pattern stained in blue (the complete images set are reported in Supplementary Figure S2).

In addition, we considered the Receiver Operating Characteristic (ROC) curves of the tissues miRNAs expression. The specificity and sensitivity of the treatment response prediction was evaluated by the Area Under the Curve (AUC) values and the 95% confidence intervals (CI). The miR-125b ROC curve data reflected the strong ability to distinguish between the responder and non-responder groups, showing an AUC of 0.9026 (95% CI, 0.7818-1.043) (Figure 3B). Regarding the other two miRNAs, they showed a good discriminating power too, although the AUC values were lower compared the one of miR-125b, respectively 0.7828 for miR-299-5p (95% CI, 0.6162–0.9494) and 0.7778 for miR-154 (95% CI, 0.5698-0.9857) (Supplementary Figure S3).

We also performed a multivariate analysis in order to exclude variables such as age, sex, type of surgery and type of treatment as confounding factors. We found that within the 11 predictive miRNAs, the expression level of miR-125b is the only one significantly correlated with the onset of acute and hematological toxicity during treatment (p-value=0.0286 and p-value=0.0475 respectively) (Supplementary Table S1).

### Serum miR-125b represents a non-invasive predictive biomarker for pCRT

**Figure 4:**
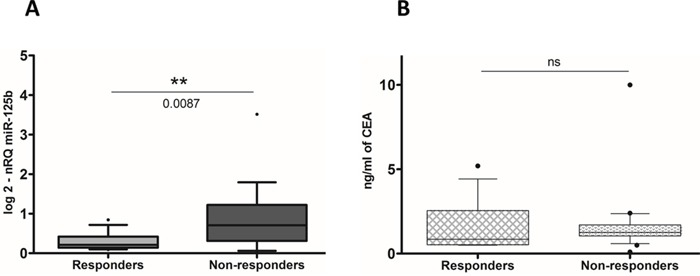
**A.** Circulating miR-125b expression levels were detectable and significantly higher in non-responders patients than in responders (nRQ, normalized Relative Quantity). **B.** Conversely, CEA level are not significantly expressed.

**Figure 5:**
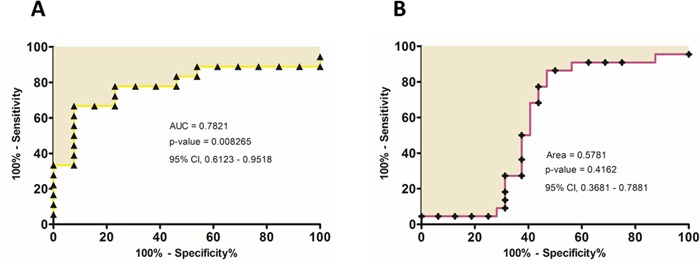
**A.** ROC curve analysis were performed based on circulating miR-125b expression level and CEA level **B.** in order to estimate their efficiency on correctly classifying patients according to the treatment response.

Based on the results showed until now, that confirm the role of miR-125b as a predictive biomarker of the pCRT patient's response, we hypothesized that higher miR-125b expression levels in non-responders could influence miR-125b expression in the serum of LARC patients. Therefore, we analyzed miR-125b serum levels in 34 LARC patients before having pCRT. Serum level of miR-125b were significantly higher in NR patients than in R (p-value=0.0087) (Figure 4A). To better compare between the miR-125b expression levels in the circulation and in the tissue, we calculated a ROC curve of the circulating miR-125b (Figure 5A). The area under curve of 0.7821 (95% CI, 0.6123 – 0.9518) reflected a good discrimination power, in terms of specificity and sensitivity. Furthermore, in the same patients, the Carcinoembryonic Antigen (CEA) serum level before pCRT showed an AUC of 0.5781 (95% CI, 0.3681–0.7881) (respectively, Figure 4B and Figure 5B) a value not sufficient to mark a clear distinction between the two groups considered (t-test, p-value=0.4235).

## DISCUSSION

The results of the present study can be summarized as follows: *i)* we found 11 differently expressed miRNAs in LARC patients, able to predict the pCRT treatment response; *ii)* among them, miR-125b shows an excellent ability to discriminate the two groups and positively correlate with the acute and hematological toxicity usually associated to pCRT; *iii)* miR-125b overexpression can be detected in the serum of LARC patients and therefore could be used as a novel non-invasive predictive biomarker of the treatment response.

Pre-operative chemoradiotherapy (pCRT) represent, to date, the best treatment option for patients with locally advanced rectal cancer. However, after pCRT the complete pathological response rate is approximately 20%, whereas in 20 to 40% of patients the response is poor or absent [7]. Indeed, the clinical question remains whether, in those patients that “*a priori*” develop tumor resistance, is possible or not to avoid unnecessary, toxic and expensive chemoradiotherapy treatment, directly performing surgery. In this landscape, the identification of predictive markers of pCRT response is one of the main priorities in LARC patients' management.

In this study, we performed microRNA expression profiling on 38 LARC tissue samples. Using microarray technology, we were able to identify 11 specific miRNAs that significantly predict the treatment response in LARC patients. Two of the identified microRNAs have been previously associated to RC. Gaedcke and colleagues reported a downregulation of miR-338-3p in a series of RCs compared to the adjacent normal mucosa. They classified miR-338-3p as rectal cancer-specific miRNA based on their miRNA profiles data regarding rectal cancer and the one previously published about colon cancer [41]. Furthermore, Svoboda and colleagues demonstrated that, two weeks upon capecitabine chemoradiotherapy-based treatment, a significant increase of the median expression of both miR-125b and miR-137 occurs in RC patients, [26]. However, although the scientific effectiveness of these data, the results we obtained could not be compared to these ones due to the different therapeutic approach used in our study to treat LARC patients.

Based on our data, miR-125b was the best predictive biomarker of the tumor response. It has been already shown by Wang *et al*. in breast cancer [38], by Shiiba *et al.* in oral squamous cell carcinoma [39] and by Haemmig *et al.* in glioblastoma [40], that this small molecule definitely plays a pivotal role in the resistance of the tumor to the therapy.

Furthermore the overexpression of miR-125b in non-responders patients is consistent with what shown in literature that demonstrated its negative correlation to proteins that stimulate apoptosis and cell cycle, such as: BAK 1, Puma, Cyclin c, Cdc25c and p53 [42]. On the other contrary, Gong *et al.*, observed a significant down-regulation of miR-125b in hepatocellular carcinoma and a correlation between its decreased expression and the reduced apoptosis rate [43]. In Gong *et al.* study which involved CRC line model, both gain- and loss- of function analyses revealed that miR-125b did not only induce spontaneous apoptosis in cancer cells, but also sensitized cancer cells to various apoptotic stimuli, including nutrient deprivation and chemotherapeutic treatment [44].

**Figure 6:**
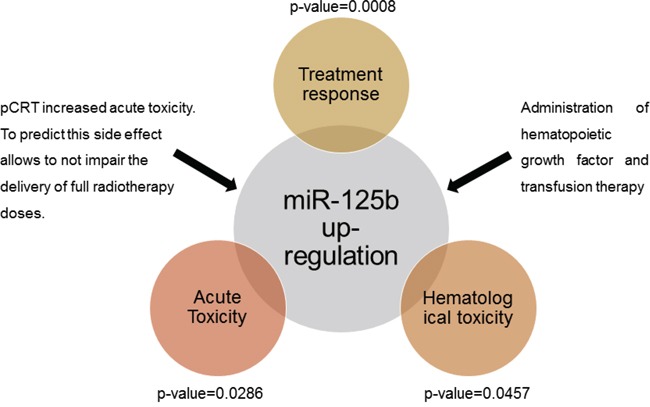
Measurement of tissue miR-125b allows a correct prediction of the patients' response to treatment and a good correlation with the manifestation of acute and hematological toxicity associated to pCRT

To the best of our knowledge, our study is the first able to show a significant positive correlation between miRNA expression and the onset of acute and hematological toxicity associated to pCRT administration in LARC setting. As reported by Berardi *et al* [45], the combined use of chemo and radiation therapy is often related to an adverse reaction increase in LARC patients, however up to now no specific markers have been associated to these effects (Figure 6).

By qRT-PCR analysis of 34 pre-operative serum samples of LARC patients, we demonstrated that the expression profile of circulating miR-125b reflects what found in tumor tissue. Non-responder patients shows a significant increased expression of miR-125b, which allows to correctly discriminate the two groups. Furthermore, we have demonstrated that the predictive power of this novel biomarker is higher than that of CEA, the only currently accepted circulating biomarker. Indeed, CEA serum levels do not differ significantly between the two groups and its ROC curve shows lower sensitivity and specificity levels. Conversely, the ROC curves of miR-125b further demonstrated the good capability of miR-125b to mark a distinction between the two groups of LARC patient (NR and R), not only at the tissues levels but also at the circulating levels. Thus, circulating miR-125b analysis could represent an innovative step-forward towards a more personalized approach in LARC patients' management based on a biological rationale. Further studies will be necessary to elucidate the mechanisms through which miR-125b is involved in the decrease of the tumor sensitivity to the pCRT.

## PATIENTS AND METHODS

### Patients

The retrospective study included consecutive patients treated, between April 2007 and October 2011, in the Department of Surgery, University of Padua, Italy. A total of 38 histopathological and 34 serum samples collected from 38 LARC patients (M/F 25/13; age 64.2 ± 8.3) were considered and included in this study, as summarized in Table 1. All the histopathological samples were retrospectively collected from the Surgical Pathology & Cytopathology Unit at the University of Padua. A series of 34 serum samples were obtained from the Surgery Unit at the University of Padua (Department of Surgery, Oncology and Gastroenterology), for 4 out of 38 patients we did not have sufficient amount of serum to perform the scheduled analysis. All the patients fulfilled the following criteria: histological confirmed primary adenocarcinoma of the rectum, tumor within 11 cm from the anal verge by proctoscopic examination, clinical stage cT3-4 and/or N0-2, resectable disease, age≥18 years, Karnofsky Performance Status ≥ 60%, and written informed consent permission. Baseline, at diagnosis, carcinoembryonic antigen (CEA) level was also determined. After pre-therapeutic staging all the patients were treated with pCRT. Patient received a total dose of at least 45 Gy to the whole pelvis at 1.8 Gy daily, 5 times per week; concomitant chemotherapy consisted in 5-Fluorouracil (5-FU) intra-venous or Capecitabine per os, with or without Oxaliplatin. The dose of radiotherapy or chemotherapy was not reduced in any of the patients. Surgery was planned 6-8 weeks after completion of chemoradiotherapy. The surgery techniques were standardized and comprise: low anterior resection (LAR); abdominoperineal resection (APR); local excision (LE) and ultralow anterior resection with colon-anal anastomosis (CAA).

### Pathological assessment and definition of tumor response

Standardized histological examination of the surgical specimens was done according to the American Joint Committee on Cancer (AJCC) guidelines. In particular, the histologic tumor response to chemoradiotherapy was assessed according to the modified tumor regression classification of *Mandard et al*. [37]. For the purpose of this study, patients were subdivided in responders (R, TRG 1-2) and non-responders (NR, TRG 3-5). Acute toxicity and Hematological toxicity adverse effects (CTCAE scale from G1 to G5) were recorded during and after treatment administration.

### Tissue samples and RNA extraction

Endoscopic tumor and normal adjacent rectal biopsies were collected from each patient before pCRT, according to a standard protocol approved by the local ethics committee. Briefly, each patient signed an informed consent for the use of these samples for research purposes. All biopsies underwent standardized histopathological examination based on hematoxylin-eosin staining of 5 μm frozen sections. Tumor specimens with≥ 70% malignant cells were considered for the experiment. Total RNA extraction, from at least 2 micro-biopsies, was performed using TRIZOL® Reagent (Invitrogen, Carlsbad, CA) following standard procedures from each endoscopic biopsy by sections of 20 μm thick. Total RNA was preserved in a final volume of 20 μl of DEPC water at -80°C with 1μl RNase Inhibitor (RNaseOUT Recombinant, 40 U/μl, Invitrogen). RNA quantity was measured on an ND-1000 spectrophotometer (NanoDrop Technologies) and quality was assessed by capillary electrophoresis with Agilent 2100 Bioanalyzer (Agilent Technologies, Inc., Santa Clara, CA). Samples with RIN> 6.5 (RNA 6000 Series Nano Chips) and samples enriched for small nucleic acid fragments with a percentage <35% (Agilent Small RNA Kit) were selected for the microarray analysis and qRT-PCR.

### Circulating RNA isolation

Blood samples were collected at the time of endoscopy, before pCRT and surgery. All samples were processed within 2–4 h after collection as follows: serum was separated by centrifugation at 3000 rpm for 15 min, divided into aliquots and immediately cryopreserved at -80°C. 200μl of human serum was thawed on ice and a fraction of small RNA were extracted using miRNeasy Serum/Plasma Kit (Qiagen). The extracted RNA was eluted in 14μL of RNAse-free water, concentration and purity were controlled by UV spectrophotometry (A260/A280 > 2.0; A260/A230 > 1.8) using Nanodrop ND-1000 (Thermo Fisher Scientific, USA). The RNA samples were immediately stored at -80°C until use. To allow for normalization of sample-to-sample variation in RNA isolation, synthetic C. elegans cel-miR-39 (synthetic RNA oligonucleotides synthesized by Invitrogen) were added to each sample before RNA isolation (at a concentration of 7 pg/pl).

### microRNA expression analysis

We used the human miRNA microarray platform Rel 12.0 (V3) manufactured by Agilent SurePrint Technology containing 866 human and 89 human viral miRNA probes. miRNA labeling, hybridization and washing were carried out according to the manufacturer's instructions. The data were filtered and rank-normalized using the ‘scale’ function from the ‘base’ package of R. All expression data have been registered in Gene Expression Omnibus (GEO) database, ID: GSE68204.

### Statistical analysis of the expression data

The statistical analysis was performed with R statistical software. Unsupervised hierarchical clustering analysis was performed with the Ward clustering method and Euclidean distance. The differential gene and miRNA expressions between R and NR were called as significant after applying the Wilcox-test (p-values <=1e-2). Multivariate analysis was carried out using the ‘aov’ function of R stats package.

### Real-time quantitative PCR (qRT-PCR)

qRT–PCR was performed as follow: 10ng of total RNA were reverse transcribed by using stem-loop RT specific primers for miR-125b (ID: 000449), miR-299-5p (ID: 000600), miR-154 (ID: 000477), small nuclear snRNA U6 (ID: 001973), and cel-miR-39 (ID: 000200), following the instruction of TaqMan MicroRNA Reverse Transcription kit (Applied Biosystems, Foster City, CA, USA). miRNA expression was measured with a specific TaqMan MicroRNA Assay and assayed on an ABI Prism 7500 System (Applied Biosystems) with cycling conditions of: 95°C for 10 min, followed by 40 cycles of 95°C for 15 s and 60°C for 60 s. The qRT-PCR was performed in triplicate.

### *In situ* RNA hybridization (ISH)

Locked nucleic acid (LNA) probes with complementarity to 22-bp sections of has-miR-125b were labeled with 5′-digoxigenin and synthesized by Exiqon (Copenhagen, Denmark). Tissue sections were digested with ISH protease 1 (Ventana Medical Systems, Milan, Italy) and ISH performed as described, with minor modifications [35]. Positive (U6; Exiqon) and negative scrambled LNA probes were used as controls. A series of 8 pre-treatment endoscopy biopsies obtained from 4 responders and 4 non-responders rectal cancers were analyzed.

### Statistical analysis

Relative expression of selected microRNA were normalized to snRNA U6 for tissue sample and with a combination of endogenous hsa-miR-16 and exogenous spike-in cel-miR-39 for serum sample. For serum samples, normalization was reached using a median normalization procedure, as previously described with minor modifications by Mitchell et al. [46]. Normalized expression was calculated using the comparative Ct method, and the fold change was expressed as 2−ΔΔCt. Differences in miR-125b, miR-299-5p, miR-154 and CEA expression level were evaluated by Wilcoxon t-test using GraphPad Prism 5.0 software. Significance (Wilcoxon t-test): *p<0.05, **p<0.01, ***p<0.001. Data were expressed as mean values ± SD. Receiver operating characteristic (ROC) curves were generated, and the area under the ROC curve (AUC) with 95% confidence intervals (CI) were calculated to assess the discriminating capability of miR-125b, miR-299-5p, miR-154 and CEA using GraphPad Prism 5.0 software.

## SUPPLEMENTARY FIGURES AND TABLE



## Abbreviations

CRC: Colorectal cancer
RC: Rectal cancer
LARC: Locally Advanced Rectal Cancer
pCRT: pre-operative chemoradiotherapy
miRNA: microRNA
5-FU: 5-Fluorouracil
CEA: Carcinoembryonic antigen
Gy: Gray
LAR: low anterior resection
APR: Abdominoperineal resection
LE: Local excision
CAA: Ultralow anterior resection with colon-anal anastomosis
CTCAE: Common Terminology Criteria for Adverse Events
TRG: Tumor Regression Grade
R: Responders
NR: Non-responders
RIN: RNA Integrity Number
FDR: False Discovery Rate
ROC: Receiver operating characteristic
AUC: Area Under Curve
CI: Confidence Intervals
ypT: pathological T stage after neoadjuvant treatments.
