# Supplementary material for: Serum miR-125b is a non-invasive predictive biomarker of the pre-operative chemoradiotherapy responsiveness in patients with rectal adenocarcinoma
Source: Oncotarget. 2016 Apr 13;7(19):28647–57. doi: 10.18632/oncotarget.8725 (PMC5053752; doi:10.18632/oncotarget.8725)
Supplement: Supplementary file 1 [file oncotarget-07-28647-s001.pdf]

Serum miR-125b is a non-invasive predictive biomarker of the pre-operative chemoradiotherapy responsiveness in patients with rectal adenocarcinoma

SUPPLEMENTARY FIGURES AND TABLES

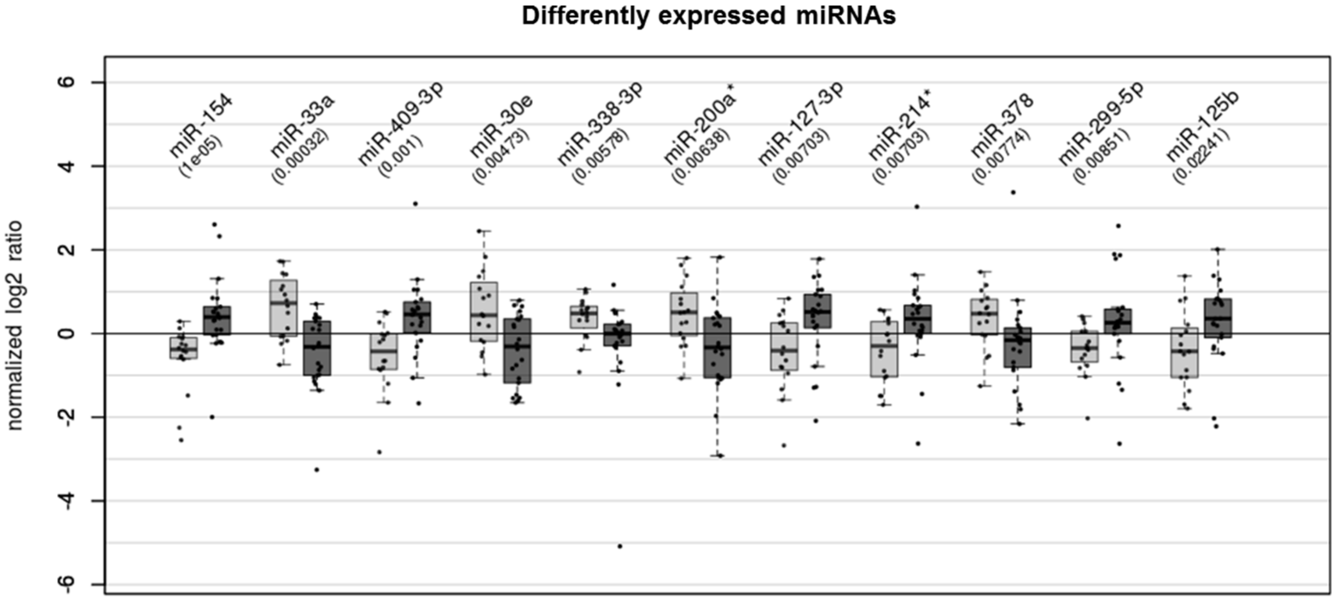

Supplementary Figure S1: Box plot resuming all miRNAs differently expressed between responders and non-responders patients (Responders, light grey; Non-responders, dark grey).

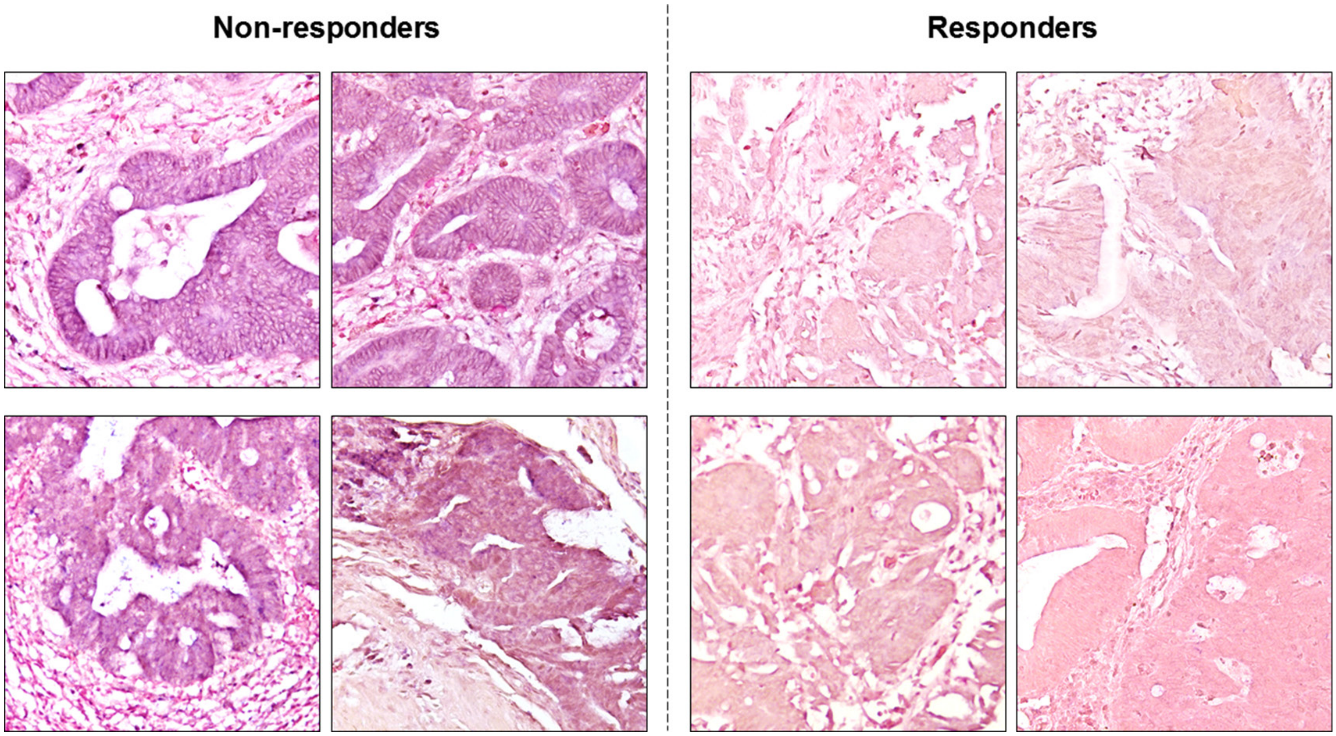

Supplementary Figure S2: *In situ* hybridization complete set of images belonging to 4 responders and 4 non-responders. It was performed to confirm miR-125b over-expression in non-responders patients. The presence of miR-125b is shown by a grainy blue cytoplasmic stain; slides counterstained in fast red. (Original magnifications 20x).

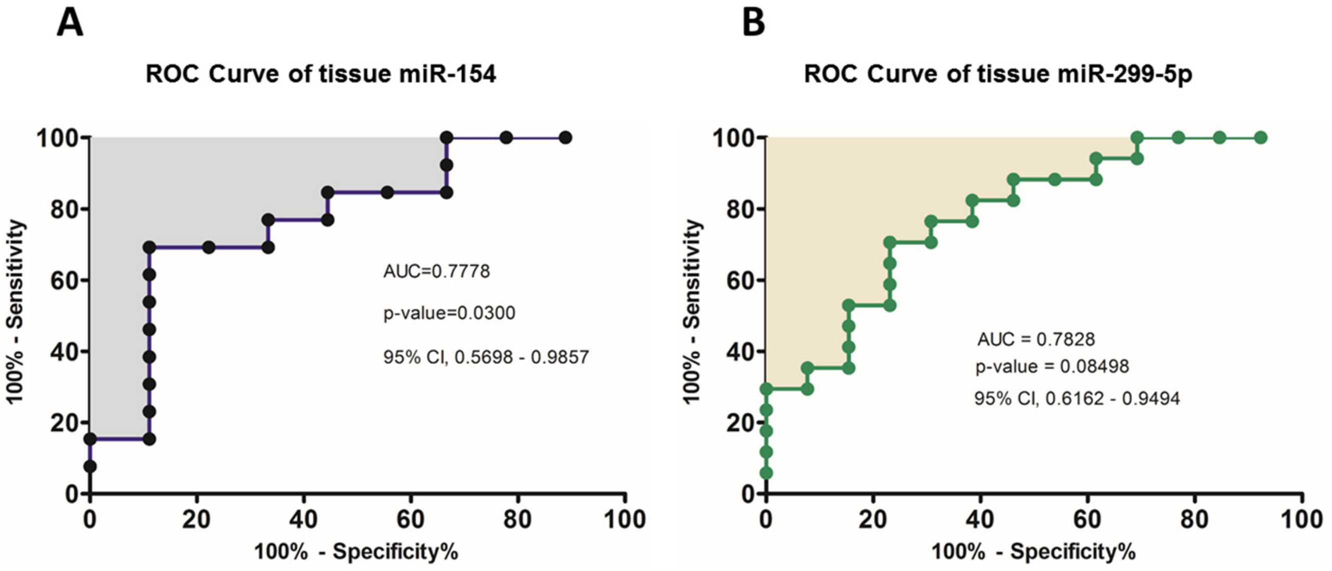

Supplementary Figure S3: A. ROC curve analysis were performed based on tissue miR-154 and B. miR-299-5p expression level shows a nearly identical discriminating power of the two groups.

Supplementary Table S1: Following multivariate analysis, among 11 selected miRNAs, miR-125b is the only one able to predict acute and hematological toxicity onset after pCRT

miRNAs	Haematological Toxicity (p-value)	Acute Toxicity (p-value)
**miR-409-3p**	0.689	0.268
**miR-299-5p**	0.645	0.416
**miR-154**	0.226	0.387
**miR-214***	0.814	0.147
**miR-127-3p**	0.861	0.422
**miR-125b**	**0.0475***	**0.0286***
**miR-30e**	**0.0112***	0.1987
**miR-200a**	**0.00324****	0.0531
**miR-378**	0.114	0.660
**miR-338-3p**	0.291	0.518
**miR-33a**	0.451	0.141

Significance (t-test): *$p<0.05$, **$p<0.01$.
